# Government intervention, industrial structure, and energy eco-efficiency: an empirical research on new energy demonstration in cities

**DOI:** 10.1038/s41598-023-46799-1

**Published:** 2023-11-09

**Authors:** Xiaoyi Zhang, Rui Zhang, Yue Wang, Meilin Zhao, Xin Zhao

**Affiliations:** School of Management, China University of Mining and Technology (Beijing), Beijing, 100083 China

**Keywords:** Energy policy, Energy efficiency

## Abstract

This study investigates the relationships among government intervention, industrial structure, and energy eco-efficiency (EE). Energy eco-efficiency was measured based on a non-radial directional distance function for 236 cities in China from 2005 to 2019. Additionally, the difference-in-difference model (DID) method and spatial econometric models were used to analyse the impact of government intervention and industrial structure on energy eco-efficiency and their spatial spill-over effects. Government intervention includes fiscal expenditures and policy orientation for new energy demonstration construction. Our results indicate that: China’s EE has a fluctuating upward trend and increased 17.85% in the period, and its spatial distribution imbalance gradually developed into a regional distribution balance. Moreover, government intervention and adjustment of the industrial structure improved urban energy eco-efficiency by 7.43% and 0.92%, respectively, which also has spatial spill-over effects in neighbouring regions. Furthermore, economic development, technological innovation, and foreign direct investment enable EE. However, urbanisation hinders the improvement of energy eco-efficiency. Finally, heterogeneity analysis showed that the policy of the new energy demonstration city has better effects on eastern and western cities in promoting EE.

## Introduction

Global warming poses an irreversible threat to human economic and social development. The consumption of fossil fuels and emission of industrial waste are the leading causes of the greenhouse effect. Using renewable energy can effectively alleviate the negative impact of fossil fuels, mitigate climate change, and improve energy security. In China, the renewable energy sector has developed rapidly owing to recent policies and incentives. From 2005 to 2006, China promulgated and implemented the Renewable Energy Law. A series of policies, including target planning, fiscal support, tariff control, and consumption guarantee, were introduced, which promoted the large-scale utilisation of renewable energy. Renewable energy consumption in China reached 454 million tons of standard coal in 2022^1^, accounting for 8.34% of the total primary energy consumption, representing an increase of 18.01% from 2021. This renewable energy development benefits China's energy transition and low-carbon economic development, substantially improves the energy structure, helps cope with climate change, and improves EE.

Energy eco-efficiency (EE) combines the economic and ecological benefits generated by energy consumption^2,3^, and can be interpreted as maximising socio-economic benefits with minimal energy consumption and ecological damage. This indicator comprehensively reflects the system efficiency of the "energy-economy-environment". In social development, a laisser-faire market economy that pursues economic interests often neglects damage to the environment, thus necessitating government intervention.

To achieve a clean energy structure and ecological civilisation construction strategy, China launched the construction of new energy demonstration cities, with the first batch of 81 cities (districts) being announced in January 2014. The construction of new energy demonstration cities allows China to explore an energy development path with Chinese characteristics, and substantially impacts EE. In this study, the construction of new energy demonstration cities was considered as the policy orientation of government intervention. Policy orientation refers to the policies and regulations issued by the government to encourage the development of new energy, including information resources, financial support, market resource allocation, development direction, talent attraction, and technological innovation.

Fiscal expenditure, a means of government intervention in the social market economy, can promote the economy and reduce emissions. Fiscal expenditure has a "multiplier effect", and fiscal input by the government can effectively promote national economic growth. However, problems with energy consumption and environmental pollution accompany economic development models. Moreover, the government's strengthening of financial investment and taxation measures in specific fields can also effectively promote technological innovation, and carbon reduction. However, it remains controversial as to whether the emission reduction effect of fiscal expenditure and the pollution caused by economic growth are balanced and coordinated, and whether this balanced effect will influence EE. The industrial structure is a major determinant that affects the intensity of resources and the degree of environmental friendliness in economic growth, and is closely related to EE^4^. EE improves when the pattern of structural change in economic development has a prominent effect on pollution. However, the change in the industrial structure caused by the simple accumulation of factors cannot improve the efficiency of resource utilisation, and may damage the environment^5^. Therefore, to effectively implement China's high-quality development strategy, it is necessary to elucidate the relationship between China's industrial structure changes and EE.

The potential contributions of this study are twofold. First, the impact of government intervention and industrial structure on EE was explored by constructing a non-radial directional distance function (NDDF) and a difference-in-difference model (DID). In the evaluation process, the EE of 236 cities in China was measured based on the NDDF. Additionally, the DID was used to identify the impact of government intervention and industrial structure on EE. Second, the spatial econometric model was used to analyse the spatial spill-over effects and regional heterogeneity of government intervention and industrial structure, and to provide policy recommendations for new energy demonstration cities.

This article is divided into seven sections. The next presents a literature review. The “Models and variables” section describes econometric models, selected sample data, and variables. The “Empirical results and analysis” section explains the analysis of the EE measurement results and benchmark regression. The “Robustness tests” section discusses parallel trend tests, superposition policy tests, and Propensity Score Matching and Difference-in-Difference (PSM-DID) tests. The sixth section, “Spatial spill-over effect and heterogeneity analysis”, presents the empirical results of the heterogeneity analysis and extended analysis of spatial spill-over effects. Finally, the conclusions and policy recommendations are discussed in the last section.

## Literature review

EE evaluation methods include the super Slacks-Based Measure Model (Super-SBM)^2^, stochastic frontier model analysis (SFA)^6^, and super-efficiency models based on NDD (Super-NDDF)^7^. An analysis of the spatiotemporal characteristics and influencing factors of EE showed that, the EE of cities in China is generally at a low level; complex fluctuations characterise the time pattern, and the EE of each city has improved to varying degrees^6^. China’s EE has significant global and local spatial agglomeration characteristics, but the spatial distribution is uneven, and there are prominent spatial effects^8^. Additionally, Wang et al.^9^ calculated and analysed the EE of the energy-enriched area of the Yellow River Basin using SFA and the Spatial Durbin Model (SDM), and found that the EE was relatively low and showed a downward trend. Innovation and industrial structure were found to be prominent factors enhancing EE in this region.

The academia has faced considerable controversy over the impact of government intervention on factor productivity. Some researchers have concluded that government intervention can achieve economic and environmental objectives by improving the impact of resource endowments on energy efficiency^10^, thus effectively improving the efficiency of the green economy^11^. However, some researchers have found that government intervention will disrupt the market by restricting private investment, reducing the efficiency of capital allocation, and inhibiting the increase in factor productivity^12^. The government intervenes in social development mainly through economic intervention and policy orientation, in which fiscal expenditure is the primary method of economic intervention. Researchers have studied the relationship between factor productivity and fiscal expenditure, finding that the emission of sulphur dioxide and other pollutants reduces with an increase in public financial expenditure^13^, which can significantly promote total factor energy efficiency^14^. Zhang et al.^15^ found that government fiscal expenditure can improve EE; however, many researchers also concluded that fiscal expenditure will negatively affect factor productivity. The inhibitory effect on green total factor productivity increases with increasing fiscal expenditure^16^. Fang et al.^17^ found that local governments may underestimate environmental protection in industrial diversification, while the decentralisation of fiscal expenditures will inhibit the improvement of EE.

By studying the impact of relevant policies and regulations with policy-oriented backgrounds on EE, researchers have found that it is difficult to meet the dual needs of improving life satisfaction and economic level by solving the conflict between the environment and energy utilisation through market mechanisms^18^. Chen et al.^7^ measured the EE of 282 cities in China using Super-NDDF and found that environmental regulation has a spatial spill-over effect in improving ecological efficiency. Cui et al.^2^ studied the impact of environmental regulation on EE based on Tobit and threshold regression models. They found that mandatory environmental regulations and market incentive environmental regulations have a more significant inhibitory effect on EE. In contrast, inhibiting voluntary environmental regulations has a time lag effect.

Regarding the research on industrial structure, many researchers have concluded that industrial structure adjustment positively affects EE. Guan and Xu^8^ found that industrial structure is the most important factor affecting EE. Industrial transformation facilitates the effective allocation of resources through factor flow and professional division of labour to effectively improve EE^19^. Liu et al.^20^ found that upgrading the regional industrial structure can considerably enhance eco-efficiency. However, some studies had different results. Meng and Zou^21^ reported that the effect of industrial structure adjustment on EE is not significant. However, increasing the proportion of industrial output will increase regional social and economic benefits, and thus inflict more severe damage to the environment. The above research shows the need for a consistent conclusion on the impact of fiscal expenditure and policy-oriented government intervention and industrial structure on EE, especially in the form of budgetary expenditure.

Researchers have found that the government can effectively guide the public to improve energy efficiency through reasonable intervention. Implementing energy policies and financial subsidies can substantially improve energy efficiency. Many countries are actively committed to implementing various policies to promote energy efficiency and establish a sustainable energy mix. The policy orientation of constructing new energy demonstration cities facilitates the utilisation of renewable energy and can effectively alleviates pollutant emissions through government support, private financing, industrial structure optimisation, and resource allocation adjustment^22,23^. Yang et al.^24^ concluded that policy orientation will encourage local governments to increase the intensity of environmental regulation for high-polluting industries and enterprises, and promote a certain amount of production factor resources transfer to policy-oriented new energy industries. The exploitation and utilisation of renewable energy can reduce dependence on traditional fossil fuel energy, enhance cities’ energy security, and promote sustainable economic, social, and environmental development^25^.

Although the spatiotemporal characteristics of EE and its impact have been studied from different perspectives, the impact of government intervention on EE and its spatial spill-over effects have yet to be explored from the perspective of new energy demonstration city policy orientation and financial expenditure. Furthermore, the influence of industrial structure adjustment on EE requires further verification. Therefore, in this study, we constructed a NDDF model considering the system of "energy-economy-environment", and then analysed the impact of policy orientation, fiscal expenditure, industrial structure, and spatial spill-over effects of government intervention on EE and its regional heterogeneity.

## Models and variables

### Econometric model

#### DID model

Government intervention is measured by fiscal expenditure and the policy orientation of constructing new energy demonstration cities. Here, we studied the impact of government intervention and industrial structure on EE. As DID can solve endogenous problems commonly faced in the existing literature^26^, the construction of new energy demonstration cities can be viewed as a "natural experiment". In this study, fiscal expenditure, policy orientation, and industrial structure were considered as core explanatory variables and a DID model was constructed to estimate the effect of government intervention and fiscal expenditure on EE. The equation for this model is as follows:

1 $$LnY_{it} = \beta_{0} + \beta_{1} du_{it} + \beta_{2} dt_{it} + \beta_{3} du_{it} \times dt_{it} + \lambda_{{1}} LnFE_{it} { + }\lambda_{{2}} LnIND_{it} { + }\lambda_{{\text{n}}} LnX_{it} + T_{{\text{t}}} + \mu_{i} + \varepsilon_{it}$$

where, $i$ and $t$ are cities and years, respectively; the explained variable $Y_{it}$ is the annual EE of each city, $du_{it}$ is a region dummy variable, and $dt_{it}$ represents the time dummy variable. The interaction coefficient $\beta_{3}$ reflects the net effect of policy orientation on EE. $FE_{it}$ is fiscal expenditure, $IND_{it}$ is industrial structure, $X_{it}$ is the control variable matrix, comprising economic development level, urbanisation level, foreign investment and technology level. $T_{t}$ is the time-fixed effect, $\mu_{i}$ is the individual-fixed effect, and $\varepsilon_{it}$ is the random disturbance term. Logarithmic processing was performed on the data of variables to reduce the influence of skewness and heteroscedasticity.

#### Spatial econometric model

A certain spatial correlation was observed because cities are not independent. Fiscal expenditure, policy orientation, and industrial structure affect regional EE and may also impact adjacent areas. Thus, in this study, spatial factors were incorporated into the model, taking government intervention and industrial structure as core explanatory variables. The spatial econometric model is as follows.

2 $$\begin{gathered} L{\text{n}}EE_{it} = \alpha_{0} + \rho WLnEE_{it} + \beta_{1} du_{it} \times dt_{it} + \beta_{2} LnFE_{it} + \beta_{3} LnIND_{it} + \beta_{n} LnX_{it} + \hfill \\ \, \xi_{1} Wdu_{it} \times dt_{it} + \xi_{2} WLnFE_{it} + \xi_{3} WLnIND_{it} + \xi_{n} WLnX_{it} + T_{t} + \mu_{i} + \varepsilon_{it} \hfill \\ \varepsilon_{it} = \gamma \varepsilon_{it} + \nu_{it} \hfill \\ \end{gathered}$$

where $W$ is the geospatial distance weight, $WLnEE_{it}$ is the spatial lag of the explained variable, $\rho$ and $\xi$ are the space-effect coefficients. $\nu_{it}$ is the error term of $\varepsilon_{it}$. When $\rho { = }\xi { = 0}$, it degenerates into a spatial error model (SEM). When $\xi { = }\gamma { = 0}$, it degenerates into a spatial lag model (SLM). When $\gamma { = 0}$, it is the SDM.

### Samples and data

In this study, 3540 balanced panel observations of 236 cities in China from 2005 to 2019 were used to investigate the impact of fiscal expenditure, new energy demonstration city construction policy orientation, and industrial structure on EE. The investigation of the construction policy orientation of new energy demonstration cities only considered the first batch of demonstration cities, and data samples at the prefecture-level were used. To ensure the robustness of the conclusion, county-level cities, districts (autonomous prefectures), and industrial park cities were eliminated from the first batch of demonstration cities established in 2014. Subsequently, 47 and 189 cities were generated in the experimental and control groups, respectively. To investigate its regional characters, the 30 provinces in China were divided into eastern, central and western regional according to geographic location. Specifically, the eastern region includes 12 provinces and cities, namely Beijing, Hebei, Tianjin, Shandong, Jiangsu, Shanghai, Zhejiang, Fujian, Guangdong, Hainan, Liaoning and Guangxi; The central region includes the nine provinces of Shanxi, Henan, Hubei, Hunan, Anhui, Jiangxi, Inner Mongolia, Heilongjiang and Jilin; The western region covers nine provinces, namely Chongqing, Sichuan, Shaanxi, Yunnan, Guizhou, Gansu, Qinghai, Ningxia and Xinjiang. Above 236 cities were divided into three regions based on their belong provinces. The data used in this study were obtained from the statistical yearbook of cities at all levels, the China City Statistical Yearbook, and the Statistical Bulletin of Social and Economic Development. The missing values were uniformly filled by interpolation.

### Variable description

#### Interpreted variables

The direction distance function (DDF) proposed by Chung et al.^27^ is extensively used in energy and environmental efficiency. However, a limitation of using the DDF is that all the input and output elements must change in the same direction. Zhou et al.^28^ further proposed the NDDF, which can effectively solve the problem of input and output factors changing in the same direction. Therefore, in the present study, the NDDF was used to measure EE. The NDDF is defined as follows^28^:

$$\overrightarrow {D} (x,y,b;g) = \sup \left\{ {W^{T} \beta :((x,y,b){ + }g \times diag(\beta )) \in T} \right\}$$

where $g = ( - g_{x} ,g_{y} , - g_{b} )$ represents the specified direction vector, $W = (W_{x} ,W_{y} ,W_{b} )$ represents the weighting vector of each input–output element, and $\beta = (\beta_{x} ,\beta_{y} ,\beta_{b} )^{T} \ge 0$ represents the variable proportion of each input and output factor.

Energy (E), capital (K), and labour (L) were taken as input factors. The gross domestic product (GDP) of each city (G) is taken as desirable output. Sulphur dioxide (S), smoke (dust) emissions (C), and wastewater discharge (P) were undesirable outputs in each city^2^. Using NDDF, the DEA model of EE of 236 cities was constructed as follows:3where the direction vector $g = ( - K, - L, - E, + G, - C, - S, - P)$, referring to the research of Liu et al.^29^, the weight matrix $W^{T} = \left( {\frac{1}{9},\frac{1}{9},\frac{1}{9},\frac{1}{3},\frac{1}{9},\frac{1}{9},\frac{1}{9}} \right)$ is given, substituting into Eq. (3), and obtaining $\beta_{j}^{ * } = (\beta_{jK}^{ * } ,\beta_{jL}^{ * } ,\beta_{jE}^{ * } ,\beta_{jG}^{ * } ,\beta_{jC}^{ * } ,\beta_{jS}^{ * } ,\beta_{jP}^{ * } )$ through linear programming, that is, the optimal solution of the slack variable of the $j$th city. The EE for each city in the corresponding year is calculated as follows:

4 $$\begin{aligned} EE_{j} = & \frac{1}{6}\left[ \begin{gathered} \frac{{G_{j} /K_{j} }}{{(G_{j} + \beta_{jG}^{ * } G_{j} )/(K_{j} - \beta_{jK}^{ * } K_{j} )}} + \frac{{G_{j} /L_{j} }}{{(G_{j} + \beta_{jG}^{ * } G_{j} )/(L_{j} - \beta_{jL}^{ * } L_{j} )}} \hfill \\ + \frac{{G_{j} /E_{j} }}{{(G_{j} + \beta_{jG}^{ * } G_{j} )/(E_{j} - \beta_{jE}^{ * } E_{j} )}} + \frac{{G_{j} /C_{j} }}{{(G_{j} + \beta_{jG}^{ * } G_{j} )/(C_{j} - \beta_{jC}^{ * } C_{j} )}} \hfill \\ + \frac{{G_{j} /S_{j} }}{{(G_{j} + \beta_{jG}^{ * } G_{j} )/(S_{j} - \beta_{jS}^{ * } S_{j} )}} + \frac{{G_{j} /P_{j} }}{{(G_{j} + \beta_{jG}^{ * } G_{j} )/(P_{j} - \beta_{jP}^{ * } P_{j} )}} \hfill \\ \end{gathered} \right] \\ { = } & \frac{1}{6}\left[ {\frac{{(1 - \beta_{jK}^{ * } ) + (1 - \beta_{jL}^{ * } ) + (1 - \beta_{jE}^{ * } ) + (1 - \beta_{jC}^{ * } ) + (1 - \beta_{jS}^{ * } ) + (1 - \beta_{jP}^{ * } )}}{{1 + \beta_{jG}^{ * } }}} \right] \\ { = } & \frac{{1 - \frac{1}{6}(\beta_{jK}^{ * } + \beta_{jL}^{ * } + \beta_{jE}^{ * } + \beta_{jC}^{ * } + \beta_{jS}^{ * } + \beta_{jP}^{ * } )}}{{1 + \beta_{jG}^{ * } }},j = 1,2,3, \ldots {,236} \\ \end{aligned}$$

In measuring EE, the labour force (L) of each city was measured based on the number of employees. Owing to the lack of city-scale energy consumption data in the statistical yearbook, provincial energy consumption data were retrieved for each city according to the light data value using a linear model without intercept^30^. The amount of capital investment was estimated using the perpetual inventory method^31^. The GDP of each city was used to express the desirable outputs. Sulphur dioxide (S), wastewater discharge (P), and smoke (dust) emissions (C) were estimated using data on industrial sulphur dioxide emissions, industrial wastewater discharges, and industrial smoke and dust emissions^2^. In this study, the impact of price factors on research was reduced by adjusting all price data in 2005.

#### Core explanatory variables

1. *Policy orientation* ($du \times dt$) The policy orientation investigated in this article is the new energy demonstration city pilot dummy variable, $du \times dt$, where $du$ is the processing variable, indicating whether the city was selected as the first batch of demonstration cities in 2014; if selected, $du{ = 1}$, otherwise $du{ = 0}$. Additionally, $dt$ is a time dummy variable; $dt{ = 0}$ before the pilot city was selected, and $dt{ = 1}$ was chosen after the selection.
2. *Fiscal expenditure* Fiscal expenditure is a government macroeconomic control measure that can intervene in economic development, pollution and carbon emission reductions, and resource allocation. By investing in new energy industry infrastructure and technological innovation, the government can continuously improve the technological innovation environment and technological service facilities, guiding the flow of innovative resources and attracting more enterprises and research institutions to increase their investment. Increasing investment in the new energy industry will accelerate the development and utilisation of new energy, reduce pollutant emissions, and promote the improvement of EE. In this study, the level of urban financial expenditure was measured as the proportion of urban general financial budget expenditure to GDP.
3. *Industrial structure* The continuous adjustment of the industrial structure can promote the rational allocation of resource elements in various industries and maintain a balance between input and output. The Thiel index is an essential indicator for measuring the reasonable allocation of industrial resource elements in cities. In this study, the Thiel index was used to measure the industrial structure. As described in Gao et al.^32^, the calculation is performed as follows:

$$\frac{{1}}{IND}{ = }\sum\limits_{{}}^{{}} {\left( {\frac{{Y_{{\text{i}}} }}{Y}} \right)} \ln \left( {\frac{{Y_{i} }}{{L_{i} }}/\frac{Y}{L}} \right)$$

where $Y_{i}$ and $Y$ represent the added value and GDP of the three industries, respectively; $L_{i}$ and $L$ are the employment and total employment of the three industries. The $IND$ value reflects a reasonable degree of industrial structure. The larger the value, the more reasonable the allocation of resources and production factors among sectors.

#### Control variables

The economic development level (PGDP) was expressed in GDP per capita. Foreign direct investment (FDI) is measured as the proportion of FDI utilised by each city in regional GDP^33^. Urbanisation level (URBAN) was measured as the ratio of the urban population to the total population. Technological innovation (TI) is measured as the proportion of employees engaged in scientific research, technical services, and geological prospecting to those employed in the unit at the end of the year. To eliminate heteroscedasticity, logarithmic processing was performed for each variable. The descriptive statistical analysis of each variable is presented in Table 1.

**Table 1:** Descriptive statistics for variables.

Symbol	Observations	Average value	Standard deviation	Minimum	Maximum
LnEE	3540	− 0.6837	0.3871	− 4.3493	0
LnFE	3540	− 1.8952	0.4398	− 3.1546	− 0.3504
LnIND	3540	1.6235	1.0740	− 0.5434	8.3866
LnPGDP	3540	10.1373	0.7872	7.8020	13.0105
LnURBAN	3540	3.8875	0.3046	2.4702	4.6052
LnFDI	3540	− 4.5921	1.3281	− 13.240	− 1.9603
LnTI	3540	− 4.2929	0.6328	− 6.4104	− 2.2659

## Empirical results and analysis

### Analysis of EE calculation results

**Figure 1:**
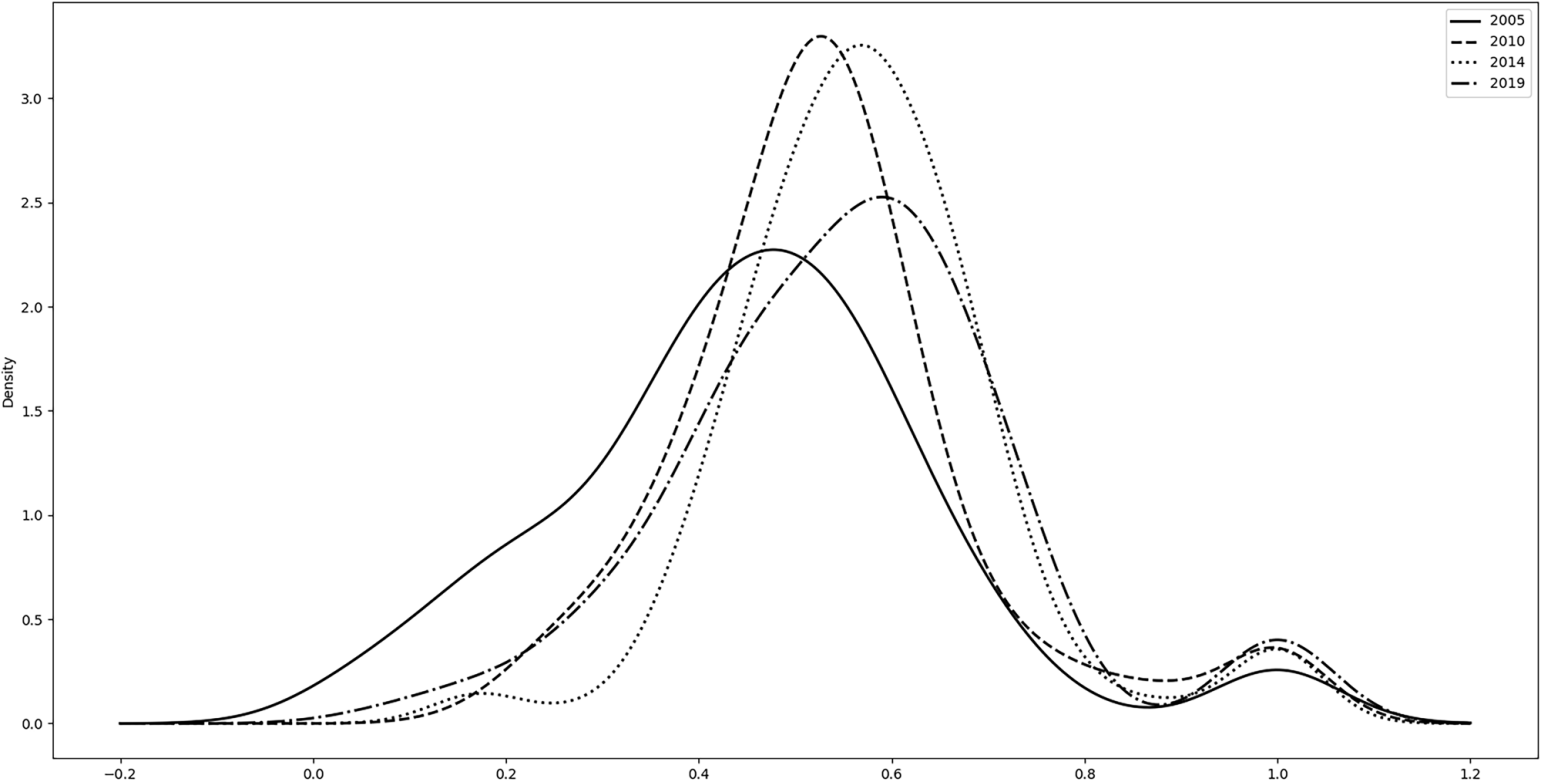
Kernel density curve of energy eco-efficiency (EE) in main years.

Equations (3) and (4) were applied to measure the EE of 236 cities in China from 2005 to 2019, and the EE kernel density curves^34^ were plotted, as shown in Fig. 1. Figure 1 shows the dynamic evolution characteristics of the EE levels of the sampled cities in 2005, 2010, 2014, and 2019. Overall, the main peak of the nuclear density curve tends to shift to the right over time, indicating that the EE level in China is constantly improving. From the perspective of the distribution position, the EE kernel density curves for 2005, 2010 and 2019 show a "double peak", with a "1 main and 2 secondary peak" feature in 2014, where the main peak is "high" and to the left, and the secondary peak is "low" and to the right (the secondary peak in 2014 refers to the right side). The part with a lower EE level has a higher peak kernel density, whereas the part with a higher EE level has a lower kernel density. This means that the current level of EE in most cities in China still needs to increase, and only a few cities have a high level of EE. The value of the main peak in each region first increased and then decreased. The width initially decreased and then increased, indicating that the absolute difference in EE among cities in China first narrowed and then expanded. The small subpeak on the left side of the kernel density curve in 2014 may owe to the new energy demonstration city policy in 2014, which made some cities start to increase the construction of renewable energy infrastructures, following energy consumption increasement and the damage to the ecological environment, Hence is energy eco-efficiency is at a low level, appearing in the left side of the main peak of the small subpeak.

**Figure 2:**
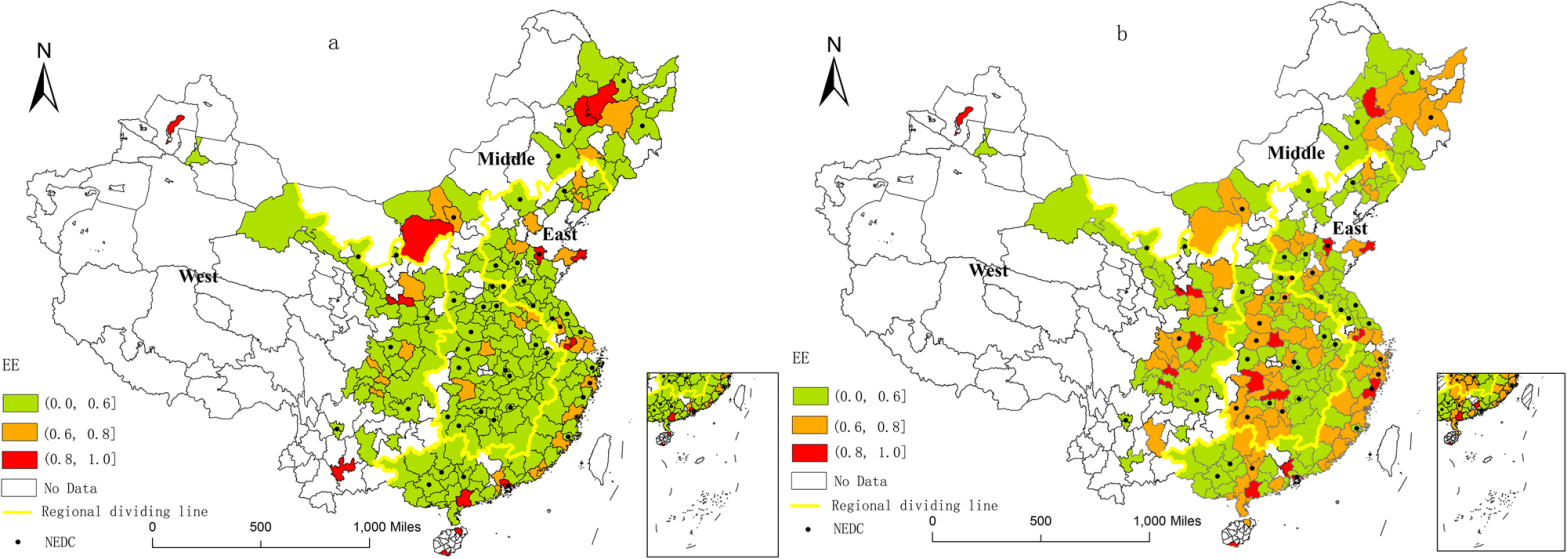
Average distribution map of energy eco-efficiency (EE). (**a**) Average distribution of EE before the policy, (**b**) Average distribution of EE after the policy. NEDC refers to the new energy demonstration city. Note: The map is produced based on the standard map with review number GS(2019)1822 downloaded from the standard map service website of the State Administration of Surveying, Mapping and Geographic Information of China, with no modifications to the base map.

The vector diagram of the average value of EE in 2005–2013 before and after the implementation of the new energy demonstration cities policy was created using ArcGIS. Combined with the breakpoint method, the average value of EE is divided into three grades: low efficiency (below 0.6), medium efficiency (between 0.6 and 0.8), and high efficiency (above 0.8), as shown in Fig. 2.

Since 2005, China’s EE has generally shown an upward trend. By 2019, it had increased by 17.85%, but still in the transition stage from low to medium efficiency. The average value of EE before and after the implementation of the policy indicates that the number of cities with different levels of EE has changed significantly. The proportion of cities with high-efficiency levels increased from 5.93% before the implementation of the policy to 7.20%. The proportion of cities with medium-efficiency levels increased from 12.71 to 40.68%, while the proportion of low-efficiency cities decreased from 81.36 to 59.75%. As shown in Fig. 2, the regional distribution of EE levels has changed. Before the implementation of the policy, the average EE in the eastern region (0.5508) was the highest, followed by that in the central (0.4887) and western (0.4828) regions. The level of EE in China has gradually increased from the western to the eastern coastal cities, and cities with medium and high-efficiency levels are mainly concentrated in the eastern region. After implementing the policy, the average EE value in all the cities increased. The region with the highest average value was still in the eastern region (0.5889), followed by the central region (0.5619), and the lowest value was observed in the western region (0.5606). Medium- and high-efficiency cities are developing towards the west and central regions, and the spatial distribution of EE has gradually achieved balanced development.

In general, medium- and high-efficiency cities have gradually developed from regional concentration to balanced regional distribution, from being concentrated in the eastern coastal areas to balanced development in the central and western regions. The western and central regions could benefit from their unique natural resources and natural gas, solar energy, clean resources such as hydroelectricity, actively cultivating green industries, and promoting win–win industry and ecology.

### Regression results and analysis

Table 2 lists the estimated results of Eq. (1). Models (1) to (4) in Table 2 introduce policy orientation, fiscal expenditure, industrial structure, and control variables in sequence. The regression model was determined to be a fixed-effects model using the Hausman test. The goodness of fit in Table 2 ranges from 0.6694 to 0.7630, indicating that the selected explanatory variables are key variables affecting EE. Among them, the goodness of fit of the regression with core explanatory variables and control variables is better, indicating that the estimated results of Model (4) can better reflect the effect of government intervention and industrial structure on EE.

**Table 2:** Difference-in-difference model (DID) regression results.

	DID			
	(1)	(2)	(3)	(4)
$du \times dt$	0.0505*** (0.0195)	0.0461*** (0.0173)	0.0385*** (0.0146)	0.0252*** (0.0076)
$du$	− 1.1841*** (0.0832)	− 1.1816*** (0.0743)	− 0.9266*** (0.0646)	− 0.3238*** (0.0341)
$dt$	0.2571*** (0.0212)	0.1951*** (0.0236)	0.1613*** (0.0203)	− 0.3115*** (0.0331)
LnFE		0.0827*** (0.0309)	0.0785*** (0.0256)	0.0491*** (0.0126)
LnIND			0.0191* (0.0106)	0.0092* (0.0055)
LnPGDP				0.3213*** (0.0246)
LnURBAN				− 0.0402** (0.0182)
LnFDI				0.0109*** (0.0020)
LnTI				0.0166*** (0.0063)
Constant	− 0.8164*** (0.0603)	− 0.5893*** (0.0928)	− 0.5842*** (0.0788)	− 3.4439*** (0.2339)
Year fixed effects	Yes	Yes	Yes	Yes
City fixed effects	Yes	Yes	Yes	Yes
Observations	3540	3540	3540	3540
$R^{2}$	0.6694	0.7070	0.7427	0.7630

The values in parentheses refer to the standard error values of the regression coefficients; ***, **, and * refer to the significance levels of 1%, 5%, and 10%, respectively.

The policy orientation ($du \times dt$) and fiscal expenditure that indicate the effect of policy have a significant positive impact on EE; both have passed the 1% significance test, indicating that government intervention with policy orientation and fiscal expenditure affects EE. Specifically, the policy orientation, financial expenditure and industrial restructuring significantly increased urban energy eco-efficiency by about 2.52%, 4.91% and 0.92%, respectively. It can be inferred that the policy formed by policy orientation is conducive to improving and supplementing the infrastructure and industrial environment on which the development of the new energy industry depends. Policy orientation often integrate innovative resources and capital. Learning and sharing effects are conducive to upgrading and innovating new energy technologies. Moreover, the optimal combination of conventional, renewable, and new comprehensive energy utilisation models can increase energy utilisation efficiency and reduce pollutant emissions, which is conducive to improving urban EE. Additionally, fiscal expenditure has a "Keynesian multiplier" effect, which promotes economic growth. Local governments have focused on green energy and environmental protection in environmental protection accountability and performance appraisal. This would promote the construction of new energy projects by increasing financial investment and support for energy conservation and environmental protection industries, actively promoting the healthy and rapid development of energy conservation and environmental protection industries, and effectively reducing pollutant emissions.

The regression coefficient of the industrial structure is significantly positive, indicating that EE can be improved by adjusting the industrial structure. This is because the allocation efficiency of resource elements can be effectively improved, the total amount of pollutants discharged in the production process can be reduced, and healthy development of the urban ecosystem and industrial economy can be achieved by coordinating the balanced development of industries. Furthermore, technological innovation, economic development level, and foreign direct investment can effectively promote the improvement of EE, indicating that in the context of industrial restructuring and government intervention, EE can be improved by attracting FDI, technological innovation, and promoting economic development. However, urbanisation has hindered EE improvement. This could be attributed to the increasing population in the city, the energy consumed in production and living, and the increase in pollution emissions, which hinders the improvement of urban EE. Moreover, urbanisation has led to the construction and use of a large amount of housing, entertainment, education, medical care, and transportation infrastructure, which has led to a sharp increase in the demand for energy and other resources. Therefore, in the planning of urban modernisation development, the government should focus on emphasising quality over quantity.

## Robustness test

### Parallel trend test

**Figure 3:**
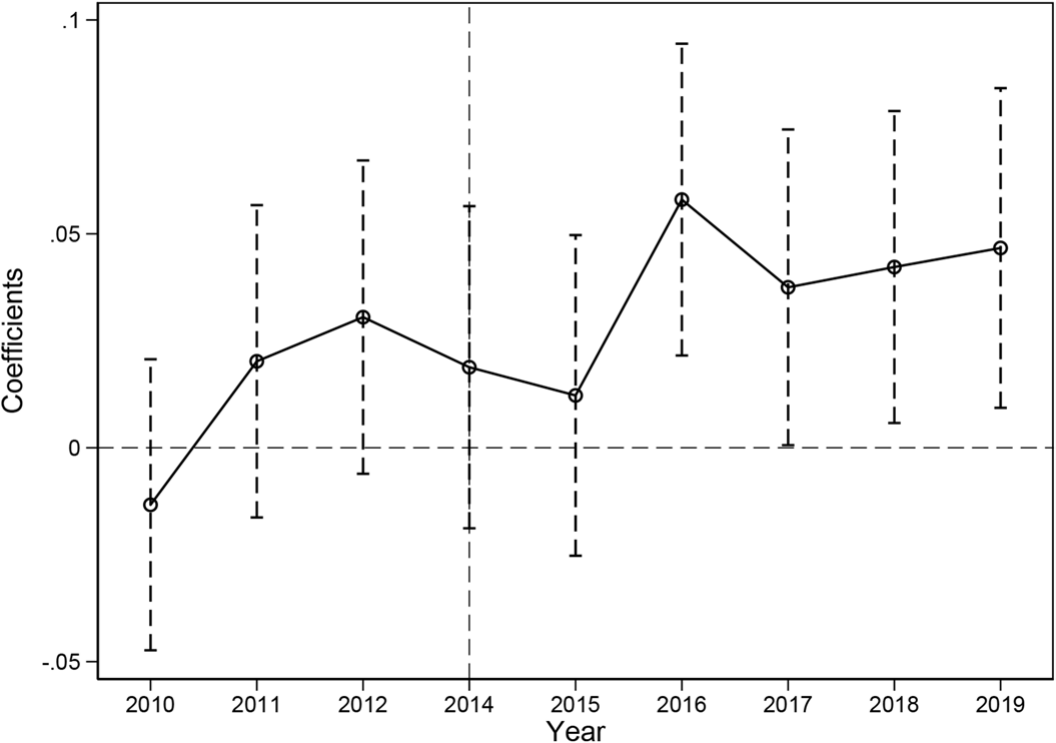
The results of the parallel trend test.

An effective assumption of the DID method is to satisfy the parallel trend^35^. Therefore, a parallel trend test was conducted using the method of Beck et al.^36^. Specifically, the regression was performed by replacing the dummy variable $dt$ in regression Eq. (1) with the dummy variable ${D}^{T}$ for each year (in which 2013 is the base period), as shown in Eq. (5). The parallel-trend test results are shown in Fig. 3. The regression coefficients of years before the implementation of the policy orientation in 2014 fluctuated around the 0 axis (the 90% confidence interval includes the 0 value). Therefore, the difference in EE between the testing and control groups is not apparent, indicating that the testing and control groups had a parallel trend before the policy was implemented. Meanwhile, the estimated coefficients pass the 10 per cent significance test from the third year of construction of the demonstration city, indicating that the impact of the pilot policy on energy eco-efficiency has a lag of three years and grows fluctuatingly thereafter. The reason could be inferred that the improvement of EE relies on industrial restructuring, and industrial restructuring has the characteristics of high investment and long cycle, coupled with the long cycle of renewable energy infrastructure investment and construction, and in the short term, it is not possible to get rid of the dependence on fossil energy consumption, and optimise the structure of energy consumption with a certain time lag, so that the impact of pilot policies on the improvement of EE has a lag.

5 $$Ln{EE}_{it}={\rho }_{0}+{\sum }_{T=-4}^{5}{\rho }_{T}{du}_{i}\times {D}^{T}+\sum Ln{X}_{it}+{\mu }_{i}+{\delta }_{t}+{\varepsilon }_{it}$$

### Superposition policy inspection

Considering that many similar or related policies between regions are implemented simultaneously or cross-wise, there is a certain overlay policy effect. Therefore, to exclude the impact of other policies in the same period, this study controlled for the impact on EE of the low-carbon city pilot and carbon trading pilot policies implemented during the sample period. Specifically, this study adds the above two policy dummy variables (the interaction term of the policy grouping dummy variable and the policy time dummy variable) to the benchmark regression to examine the causality between policy orientation and EE after controlling for other policy disturbances. Table 3 presents the corresponding estimated results. Models 5 and 6 are regression results that only contain policy variables, and Models 7 and 8 are the estimated results after adding control variables. According to Table 3, after adding other relevant policy variables, the estimated coefficient of $du \times dt$ remains significantly positive, which is the same as the result of the benchmark regression. This shows that, after considering the impact of the above policies, government intervention and industrial structure still significantly affect EE.

**Table 3:** Superposition policy test.

	(5)	(6)	(7)	(8)
$du \times dt$	0.0487** (0.0203)	0.0379* (0.0202)	0.0256*** (0.0078)	0.0224*** (0.0078)
Low carbon	0.0031 (0.0210)		0.0007 (0.0081)	
Carbon trading		− 0.1644*** (0.0279)		− 0.0442*** (0.0115)
LnFE			0.0484*** (0.0129)	0.0490*** (0.0128)
LnIND			0.0191* (0.0056)	0.0094* (0.0056)
LnPGDP			0.3348*** (0.0253)	0.3106*** (0.0260)
LnURBAN			− 0.0440** (0.0188)	− 0.0589*** (0.0191)
LnFDI			0.0115*** (0.0021)	0.0109*** (0.0021)
LnTI			0.0234*** (0.0058)	0.0227*** (0.0058)
Constant	− 0.8253*** (0.0634)	− 0.8325*** (0.0624)	− 3.5386*** (0.2407)	− 3.2521*** (0.2508)
Year fixed effects	Yes	Yes	Yes	Yes
City fixed effects	Yes	Yes	Yes	Yes
Observations	3540	3540	3540	3540
$R^{2}$	0.6554	0.6590	0.7656	0.7667

The values in parentheses refer to the standard error values of the regression coefficients; ***, **, and * refer to the significance levels of 1%, 5%, and 10%, respectively.

### PSM-DID test

**Figure 4:**
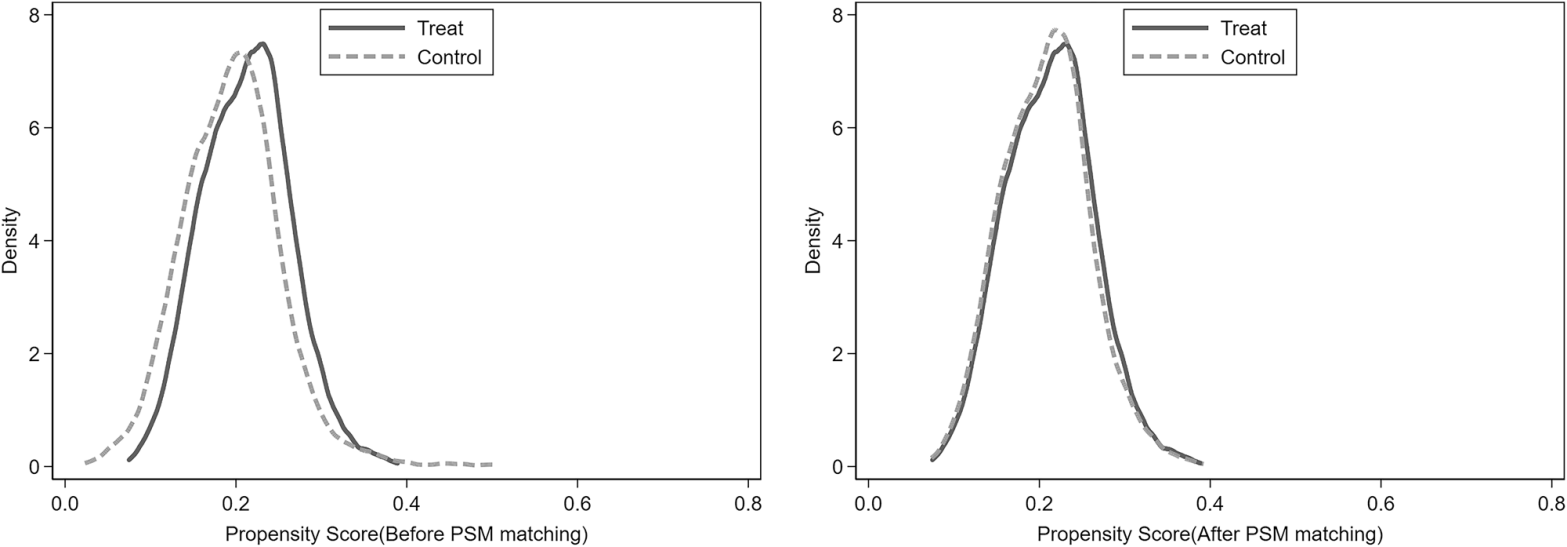
Kernel density distribution comparison of propensity score values before and after kernel matching.

In the present study, technological innovation, economic development, urbanisation level, foreign investment, fiscal expenditure, and industrial structure were used as matching variables, and the neighbour 1:1 method for matching, the matching effect is shown in Fig. 4. After the abovematching treatment, the probability distribution of tendency scores in the experimental group and the control group has basically tended to be consistent. It shows no significant systematic error, which is meeting the hypothesis of the PSM model. Regression estimation was performed as described in Eq. (1), and Table 4 shows the regression estimation results of PSM-DID.

**Table 4:** PSM-DID regression.

	PSM-DID			
	(9)	(10)	(11)	(12)
$du \times dt$	0.0528*** (0.0204)	0.0499*** (0.0174)	0.0419*** (0.0147)	0.0260*** (0.0078)
$du$	− 1.1862*** (0.0881)	− 1.1830*** (0.0757)	− 0.9287*** (0.0657)	− 0.3398*** (0.0357)
$dt$	0.2661*** (0.0221)	0.1950*** (0.0237)	0.1609*** (0.0203)	− 0.3372*** (0.0352)
LnFE		0.0837*** (0.0310)	0.0796*** (0.0257)	0.0504*** (0.0130)
LnIND			0.0189* (0.0106)	0.0091* (0.0057)
LnPGDP				0.3441*** (0.0262)
LnURBAN				− 0.0415** (0.0188)
LnFDI				0.0114*** (0.0021)
LnTI				0.0172*** (0.0065)
Constant	− 0.8244*** (0.0627)	− 0.5873*** (0.0930)	− 0.5813*** (0.0789)	− 3.6564*** (0.2487)
Year fixed effects	Yes	Yes	Yes	Yes
City fixed effects	Yes	Yes	Yes	Yes
Observations	3528	3528	3528	3528
$R^{2}$	0.6524	0.7042	0.7402	0.7637

The values in parentheses refer to the standard error values of the regression coefficients; ***, **, and * refer to the significance levels of 1%, 5%, and 10%, respectively.

Models (9) to (12) in Table 4 introduce policy orientation, fiscal expenditure, industrial structure and control variables orderly, respectively. The regression coefficient is still significant at the 10% level and the sign is positive, indicating that the improved PSM-DID model results of the DID regression model are further improved. Therefore, supporting the industrial structure and government intervention has positive significance in promoting the improvement of EE.

## Spatial spill-over effect and heterogeneity analysis

### Spatial spill-over effect

The general and specific Moran's I tests showed that the explained variables had a positive spatial autocorrelation, indicating the spatial layout of high-high and low-low clustering. A robust Lagrangian multiplier test (R-LM) and Lagrange multiplier (LM) test were performed to obtain a better fitting effect. LM test results significantly reject the null hypothesis, but the R-LM (error) failed the test^37,38^, and LM (lag) > LM (error), R-LM (lag) > R-LM (error), indicating that the SLM should be selected^39^. Furthermore, the Hausman test supported the fixed effects model. According to the $R^{2}$ and Log-likelihood values of the SDM, SAR, and SEM models in Table 5, combined with the Hausman test results, it is more reasonable to choose the SLM under individual fixed effects for empirical testing.

**Table 5:** Spatial spill-over effect regression results.

	(13)	(14)	(15)
	SDM	SAR	SLM
$du \times dt$	0.0327*** (0.0092)	0.0229*** (0.0085)	0.0248*** (0.0078)
LnFE	0.0659*** (0.0156)	0.0262* (0.0148)	0.0598*** (0.0138)
LnIND	0.0108* (0.0066)	0.0121** (0.0061)	0.0090* (0.0056)
LnPGDP	0.4354*** (0.0299)	0.0841*** (0.0122)	0.3171*** (0.0271)
LnURBAN	− 0.0579*** (0.0221)	− 0.0340* (0.0205)	− 0.0344* (0.0187)
LnFDI	0.0212*** (0.0031)	0.0177*** (0.0022)	0.0123*** (0.0021)
LnTI	0.0266*** (0.0079)	0.0221*** (0.0071)	0.0178*** (0.0067)
$\rho$/$\gamma$	0.7115*** (0.0463)	0.5793*** (0.0502)	0.8798*** (0.0251)
${\delta }^{2}$	0.0113*** (0.0003)	0.0098*** (0.0002)	0.0081*** (0.0002)
Log-likelihood	2902.0678	3162.7401	3473.7738
Observations	3540	3540	3540
$R^{2}$	0.1390	0.1504	0.1401

The values in parentheses refer to the standard error values of the regression coefficients; ***, **, and * refer to the significance levels of 1%, 5%, and 10%, respectively.

According to the SLM model results in Table 5, the regression coefficients of policy orientation, fiscal expenditure, and industrial structure on urban EE are all significantly positive, which shows that industrial structure and government intervention have significantly improved the EE effect. Moreover, the spatial autoregressive coefficient $\rho$ is significantly positive, indicating that EE has a significant endogenous spatial interaction effect among cities. The improvement of energy efficiency in this region can drive the improvement of surrounding areas and form a high–high agglomeration state in space. Specifically, the industrial structure and government intervention behaviour in a region will directly affect the local EE and indirectly affect the EE of neighbouring areas and produce a feedback effect, which will eventually further change the industrial structure and the actual impact of government intervention behaviour on local EE. LeSage and Pace^40^ proposed that the influence of independent variables on dependent variables can be divided into direct, indirect, and total effects. We conducted a decomposition analysis based on the spatial lag model under individual fixed effects^37^, as shown in Table 6.

**Table 6:** Decomposition of space effects.

	Direct effect	Indirect effect	Total effect
$du \times dt$	0.0233*** (0.0088)	0.0321** (0.0134)	0.0554*** (0.0214)
LnFE	0.0262* (0.0145)	0.0359* (0.0213)	0.0621*(0.0351)
LnIND	0.0122* (0.0064)	0.0170* (0.0098)	0.0292* (0.0158)
LnPGDP	0.0844*** (0.0122)	0.1156*** (0.0227)	0.2000*** (0.0285)
LnURBAN	− 0.0342* (0.0198)	− 0.0474* (0.0295)	− 0.0816* (0.0483)
LnFDI	0.0178*** (0.0021)	0.0247*** (0.0061)	0.0425*** (0.0074)
LnTI	0.0228*** (0.0070)	0.0316*** (0.0119)	0.0544*** (0.0181)

The values in parentheses refer to the standard error values of the regression coefficients; ***, **, and * refer to the significance levels of 1%, 5%, and 10%, respectively.

According to the decomposition results of the spatial lag model in Table 6, the direct effect of policy orientation, fiscal expenditure, and industrial structure on the EE of the city is positive. It passed the significance test, confirming that government intervention and industrial structure promote EE in the region. Moreover, the spatial spill-over effects of government intervention and industrial structure were significantly positive, indicating that fiscal expenditure, policy orientation, and industrial structure improve the EE of the region as well as that of neighbouring regions. The construction of demonstration cities is based on the market mechanism. It is oriented by government intervention, which could encourage enterprises to implement technological innovation, rationally allocate resource elements in various industries, exert technological innovation and demonstration effects, and promote the rational use and mutual flow of high-quality resource elements among regions. Furthermore, through knowledge diffusion and technological spill-over, a good demonstration effect and space radiation effect on adjacent areas can promote the "learning effect" in adjacent areas, thereby reducing the difficulty of EE improvement.

### Analysis of regional heterogeneity

The impact of government intervention and the industrial structure of cities in different geographical locations on EE may differ. Therefore, we divided the research sample into three regions in the east, middle, and west and examined the impact of fiscal expenditure, policy orientation, and industrial structure on urban EE under location heterogeneity. Table 7 presents the estimated results of the spatial-lag model.

**Table 7:** Regional heterogeneity analysis regression results.

	East	Middle	West
$du \times dt$	0.0940*** (0.0270)	0.0004 (0.0110)	0.1046*** (0.0405)
LnFE	0.0964*** (0.0336)	− 0.0265 (0.0214)	− 0.0522 (0.0361)
LnIND	0.0389*** (0.0134)	− 0.0081* (0.0043)	0.0009 (0.0198)
$\rho$	0.3769*** (0.0748)	0.4280*** (0.0743)	0.4165*** (0.0830)
Policy direct effect	0.0952*** (0.0224)	0.0008 (0.0114)	0.1071** (0.0419)
Policy indirect effect	0.0586** (0.0231)	0.0003 (0.0088)	0.0762** (0.0384)
Policy total effect	0.1538*** (0.0400)	0.0011 (0.0200)	0.1833** (0.0738)
Control variable	Yes	Yes	Yes
Log-likelihood	614.1524	1488.4396	241.9661
Observations	1470	1425	645
$R^{2}$	0.1598	0.1402	0.0851

The values in parentheses refer to the standard error values of the regression coefficients; ***, **, and * refer to the significance levels of 1%, 5%, and 10%, respectively.

During the study period, the policy orientation coefficients of the demonstration cities were all positive, which verified that policy orientation is conducive to promoting the improvement of the EE of the region. The policy orientation of western and eastern cities passed the significance test, showing direct effects. The spatial spill-over effects of western and eastern cities account for 36.23% and 39.73% of their total effects, respectively, while the driving effects of policy orientation of the central cities were not significant. Fiscal expenditure significantly impacted EE in eastern cities rather than the west and central regions. Additionally, although the industrial structure can substantially promote the EE of eastern cities, it had no significant effect on western cities and had an inhibitory effect on the EE of central cities.

This regional difference may be because the central region has a high endowment of coal resources and an incomplete industrial structure. The extensive economic growth model causes the secondary industry to account for a substantial proportion, and there are many high-energy-consuming and high-polluting enterprises. Financial expenditure and policy orientation make it challenging to adjust the energy structure dominated by traditional fossil fuel energy in the short term, which has an impact on EE. Additionally, the regional economic development model has not fully transformed from "resource factor promotion" to "green development driving" due to the weak local technology foundation, the long-term output cycle of R&D input and results, and the long-term mechanism of R&D support is not apparent. Factor productivity and technological levels are not increased as planned and even hinders the improvement of EE.

By relying on solar energy, wind energy, and natural gas resources, the western region actively supports the green industry. It promotes EE via the spatial transfer of capital, technology, and advanced concepts brought about by policy guidance. The policy benefits from the new energy demonstration construction pilots have attracted high-tech projects transferred from the central and eastern regions, and the technological spill-over effects of these projects have led to corresponding improvements in the management capabilities and technical levels of the western regions, thereby improving EE in western regions. The east is a relatively developed region with a reasonable industrial structure. It could take good use of the dominant position of a developed economy and a good industrial foundation, actively responding to policy orientation, consciously innovating green technologies, accelerating scientific and technological research and development, vigorously developing clean energy, and improving green production capacity.

## Conclusions and recommendations

Studying the internal relationship between industrial structure, government intervention behaviour, and EE in China has important implications for developing new energy, reducing pollutant emissions, and realising sustainable development. In this study, we selected panel data from 236 cities in China from 2005 to 2019, constructs the NDDF model to calculate the EE of each city, and establishes a DID model to empirically verify the effect of policy orientation, fiscal expenditure, and industrial structure on EE. The spatial impact and regional heterogeneity of government intervention and industrial structure on EE were analysed using a spatial econometric model. The research results show that China's EE is at a low level and shows an increasing trend during the research period, with a spatial distribution grid of low–low and high–high concentrations. The policy orientation, financial expenditure and industrial restructuring adjustment significantly increased urban energy eco-efficiency by about 2.52%, 4.91% and 0.92%, respectively, and industrial structure and government intervention have a positive spatial spill-over effect on EE improvement. Its spatial spill-over effect is transmitted between regions through the "learning effect" and "demonstration effect", thus promoting the improvement of regional EE. Additionally, there is spatial heterogeneity in this spill-over effect, and the driving impact of policy orientation on eastern and western cities is stronger. Foreign direct investment, economic development, and technological innovation can help improve EE, while urbanisation can inhibit the improvement of EE.

Based on the findings of this study, the following policy recommendations are proposed to promote the advancement of China's EE and promote the coupled development of energy, economy, and environment:

1. Strengthen policy guidance and financial support for high-tech and environmental protection and energy-saving industries; actively exert government intervention and industrial structure effects to cultivate new economic growth; and promote the advancement of EE using foreign direct investment and technological innovation. The government should take policy as guidance and financial support to encourage the development of strategic emerging industries in an overall way, promote the sustainable development of resource elements to industries such as low energy consumption, low pollution, high value-added environmental protection and energy conservation, high-tech and other sectors, reasonably promote the optimisation and upgrading of industrial structure, and promote the sustainable development of the green economy. Moreover, the government should advance the coordinated development of the business environment, technological innovation, and sustainable urbanisation. The government should improve the business environment and compensation mechanism for technological innovation, strengthen the construction of public research and development institutions and experimental platforms, and make more efficient and standardised use of funds from various sources, such as foreign capital. Additionally, it promotes the innovation and application of green technology in urbanisation and achieves the high-quality, coordinated development of urbanisation and the environment.
2. Actively promote the construction of new energy demonstration cities, strengthen the policy orientation effect of government intervention, and provide greater policy guarantees for the country’s overall new energy development. On the one hand, give full play to the policy-oriented resource allocation function, continue to promote the construction of new energy demonstration cities, strengthen the spatial spill-over effect of the construction of demonstration cities, improve the radiation scope and influence of the development of new energy cities, and achieve the high-quality development of new energy. But on the other hand, they are continuously optimising demonstration policies, strengthening policy systems such as intellectual property protection, ensuring reasonable monopoly income from innovation achievements of innovation subjects, and laying a policy foundation for constructing a strong new energy city and consummating the innovation environment.
3. Optimise the industrial structure according to local conditions, take advantage of the spatial spill-over effect of high-energy-efficiency regions, strengthen technical exchange and resource sharing, accelerate the coordinated development of EE regions, and jointly improve EE. First, it is necessary to make good use of the radiation effect of EE, strengthen inter-regional technical exchanges and resource sharing, and promote benign interactions and coordinated development between cities. Second, owing to the different characteristics of the city itself, the influence of government intervention and industrial structure on EE will have regional heterogeneity. Therefore, give full play to the advantages of resource endowment and implement a demonstration city construction plan according to local conditions.

The limitations of this study are as follows: First, this study focussed on China, which may have more reference value for developing countries but is less applicable for developed countries. Second, city-level macro-data research was used but the enterprise-level micro-angle was not considered, which has certain limitations.

## Data Availability

The datasets used and/or analyzed during the current study available from the corresponding author on reasonable request.
